# Effectiveness of everolimus for sporadic renal angiomyolipoma with inferior vena cava thrombus: A case report

**DOI:** 10.1016/j.eucr.2023.102520

**Published:** 2023-08-02

**Authors:** Kosuke Iwatani, Jun Miki, Fumihiko Urabe, Takafumi Yanagisawa, Takashi Hatano, Takahiro Kimura

**Affiliations:** a Department of Urology, The Jikei University School of Medicine, Kashiwa Hospital, Chiba, Japan; b Department of Urology, The Jikei University School of Medicine, Tokyo, Japan; c Department of Urology, Seirei Yokohama Hospital, Kanagawa, Japan

**Keywords:** Sporadic renal angiomyolipoma, Everolimus, Inferior vena cava thrombus, Laparoscopic nephrectomy

## Abstract

Sporadic Renal Angiomyolipoma (AML) is commonly managed through transcatheter arterial embolization (TAE) or surgical intervention. In this report, we present a remarkable case of tumor regression from Novick Classification level 2 to level 1 in a 54-year-old male with sporadic renal AML and inferior vena cava (IVC) involvement, treated with the administration of everolimus. Subsequently, laparoscopic nephrectomy without cavectomy was performed. Notably, the patient did not present with tuberous sclerosis complex (TSC). Our findings highlight the potential of everolimus in the treatment of sporadic AML, even in cases unrelated to TSC, offering a viable alternative to invasive therapeutic approaches.

## 1 Introduction

Renal angiomyolipoma (AML) is a benign mesenchymal tumor composed of a mixture of blood vessels, smooth muscle, and adipose tissue. While sporadic AML are more common, this tumor can also be associated with tuberous sclerosis complex (TSC). Although often asymptomatic, larger AMLs present a potential risk of rupture, necessitating therapeutic intervention such as transcatheter arterial embolization (TAE) or surgical resection.^1^ In this study, we present a notable case of a large sporadic AML with inferior vena cava (IVC) thrombus, wherein the administration of everolimus prior to surgery led to remarkable regression of tumor size.

## 2 Case presentation

**Fig. 1:**
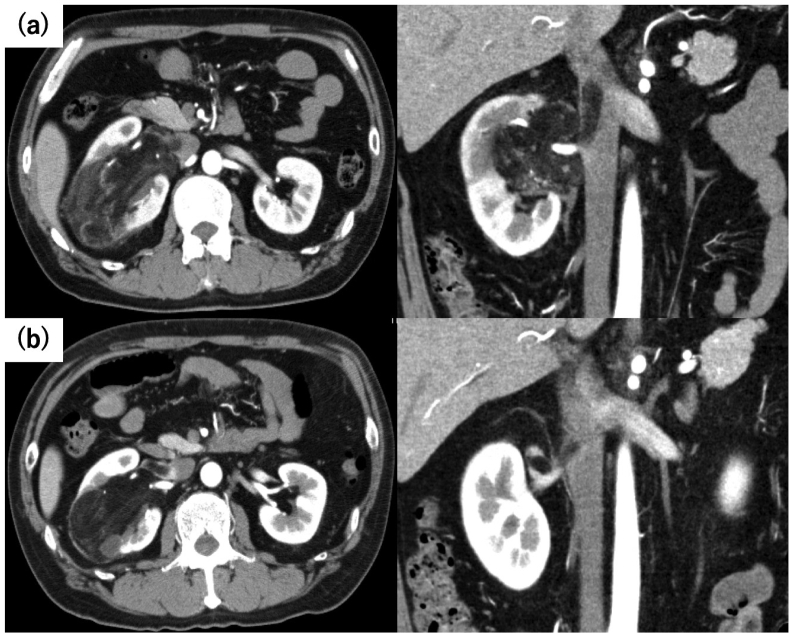
Contrast agent CT scan; Tumor and IVC thrombus (a) Pre-treatment (b) 3 months after everolimus administration.

A 54-year-old male presented with macrohematuria and was found to have a large right renal tumor measuring 110 mm, accompanied by a tumor thrombus in the right renal vein extending into the IVC (Novick Classification level 2). Physical examination revealed no other abnormalities, and there was no significant family history. Given the associated risk of pulmonary embolization due to necrotic tissues, TAE was deemed inappropriate. Due to the potential risk of surgical resection, we administered everolimus 10mg in expectation of tumor shrinkage. There were no adverse effects observed. After three months of therapy, a significant reduction in tumor diameter to 80mm was observed, along with regression of the IVC thrombus (Fig. 1).

### 2.1 Surgical procedure

Following the successful preoperative therapy, a laparoscopic nephrectomy and thrombectomy were performed. To ensure safety and simplicity, our surgical approach involved temporary clamping of the proximal IVC above the tumor, the distal IVC at the renal vein, and dissection of the right renal vein. By incising the right renal vein, the tumor was successfully extracted as a cohesive mass with the right kidney. Monitoring with ultrasound sonography confirmed complete tumor removal. The thrombus did not adhere to the vein wall, and no fibrin clot was observed. The operation lasted 5 hours and 28 minutes, with a blood loss of 200ml and no complications. The patient was discharged on the sixth postoperative day and has remained recurrence-free during the 5-year follow-up period.

### 2.2 Histopathological diagnosis

Histopathological examination of the primary tumor confirmed the diagnosis of angiomyolipoma. The tumor displayed abundant fat cells, and some vessels exhibited thickened walls. The tumor margin was negative, and no necrotic tissue was present. Due to the patient's request, genetic testing was conducted. Although there were no significant abnormalities in his genetic test.

## 3 Discussion

This report presents the first evidence of the efficacy of everolimus in the treatment of sporadic renal angiomyolipoma (AML) without tuberous sclerosis complex (TSC) genetic defects, while avoiding the need for cavectomy in cases involving inferior vena cava (IVC) thrombus.

First, everolimus showed potential as a treatment for sporadic AML cases unrelated to TSC. Everolimus is guideline-endorsement treatment in patients with TSC-related AML. Several case reports showed the efficacy of everolimus even for a large AML.^2^ In TSC patients, 85% of them have TSC1 and TSC2 genetic defects, leading to the deficiency of a protein that regulates mTORC1 activation.^3^ Everolimus directly inhibits mTORC1 and disrupts TSC-AML cell growth through the mTOR pathway.

In contrast, the molecular structure of sporadic AML remains not well-proven. In general, sporadic AML is characterized by mutations in the TSC2 gene alone, leaving uncertainty regarding the effectiveness of mTOR inhibitors.^4^ However, there have been reports of significant reduction in sporadic AML with the administration of rapamycin, which is also an mTOR inhibitor.^5^ Furthermore, a Phase II randomized controlled trial investigating the tumor shrinkage effect of Everolimus on sporadic AML demonstrated that 58% of patients achieved a reduction of 25% or more. However, 40% of patients were unable to continue the treatment due to side effects, raising concerns about its tolerability.^6^

Second, a valuable surgical strategy involves incising the renal vein and removing the tumor without cavectomy in cases of AML with IVC thrombus. While 77 cases of AML with IVC thrombus have been reported, irrespective of TSC association, all cases required cavectomy. Previous reports indicate that AML generally does not adhere to the vessel wall, and fibrin clots are rarely observed.^7^ Our surgical technique allowed for shorter IVC clamp times, eliminated the need for unnecessary IVC sutures, and potentially reduced complications and patient stress. Furthermore, there have been reports that administration of sirolimus as a pre-surgical treatment for TSC-related AML resulted in a reduction of AML size by 38–95% and allowed for safe partial nephrectomy.^8^ Given that many sporadic AMLs are benign in nature, preoperative medication aiming for less invasive and organ-preserving treatment is reasonable.

However, caution should be exercised regarding the long-term administration of everolimus in patients with IVC thrombus. Incomplete cytoreduction may result in the peeling off of necrotic tissue, which can embolize the pulmonary vein. The prolonged use of drugs necessitates careful consideration to prevent this outcome.

There is potential for everolimus to exhibit good efficacy in treating sporadic AML. Guidelines for sporadic AML remain uncertain, in comparison to invasive therapies such as TAE or surgical interventions, everolimus may be considered a potential therapeutic option. Future discussions should delve into the possibility of many sporadic AML cases exhibiting similar positive responses to everolimus treatment.

## 4 Conclusion

Everolimus shows promising potential as a non-invasive treatment option for sporadic AML. Our surgical approach provides a safe and efficient method for managing AMLs with IVC thrombus. Continued investigation and discussions are necessary to expand our understanding and refine the management strategies for sporadic AML.

## Declaration of competing interest

None declared.

## References

[1] Fernández-Pello S., Hora M., Kuusk T. (2020). Management of sporadic renal angiomyolipomas: a systematic review of available evidence to guide recommendations from the European association of urology renal cell carcinoma guidelines panel. Eur Urol Oncol.

[2] Ikarashi D., Mue Y., Shiomi E. (2017). Efficacy of everolimus for treating renal angiomyolipoma with inferior vena cava thrombus associated with tuberous sclerosis: a case report. Urol Case Rep.

[3] Northrup H., Aronow M.E., Bebin E.M. (2021). Updated international tuberous sclerosis complex diagnostic criteria and surveillance and management recommendations. Pediatr Neurol.

[4] Kenerson H., Folpe A.L., Takayama T.K. (2007). Activation of the mTOR pathway in sporadic angiomyolipomas and other perivascular epithelioid cell neoplasms. Hum Pathol.

[5] Pleniceanu O., Omer D., Azaria E. (2018). mTORC1 inhibition is an effective treatment for sporadic renal angiomyolipoma. Kidney Int Rep.

[6] Geynisman D.M., Kadow B.T., Shuch B.M. (2020). Sporadic angiomyolipomas growth kinetics while on everolimus: results of a Phase II trial. J Urol.

[7] Kheir P., Abdessater M., El Khoury J. (2020). Renal angiomyolipoma with IVC thrombus: a case report. Int J Surg Case Rep.

[8] Staehler M., Sauter M., Helck A. (2012). Nephron-sparing resection of angiomyolipoma after sirolimus pretreatment in patients with tuberous sclerosis. Int Urol Nephrol.

